# Methodology for the Automated Visual Detection of Bird and Bat Collision Fatalities at Onshore Wind Turbines

**DOI:** 10.3390/jimaging7120272

**Published:** 2021-12-09

**Authors:** Christof Happ, Alexander Sutor, Klaus Hochradel

**Affiliations:** Institute of Measurement and Sensor Technology, UMIT–Private University for Health Sciences, Medical Informatics and Technology, 6060 Hall in Tyrol, Austria; alexander.sutor@umit-tirol.at (A.S.); klaus.hochradel@umit-tirol.at (K.H.)

**Keywords:** bird, bat, automatic fatality detection, camera system, NIR, LWIR, image processing, background subtraction, region based, structural similarity

## Abstract

The number of collision fatalities is one of the main quantification measures for research concerning wind power impacts on birds and bats. Despite being integral in ongoing investigations as well as regulatory approvals, the state-of-the-art method for the detection of fatalities remains a manual search by humans or dogs. This is expensive, time consuming and the efficiency varies greatly among different studies. Therefore, we developed a methodology for the automatic detection using visual/near-infrared cameras for daytime and thermal cameras for nighttime. The cameras can be installed in the nacelle of wind turbines and monitor the area below. The methodology is centered around software that analyzes the images in real time using pixel-wise and region-based methods. We found that the structural similarity is the most important measure for the decision about a detection. Phantom drop tests in the actual wind test field with the system installed on 75 m above the ground resulted in a sensitivity of 75.6% for the nighttime detection and 84.3% for the daylight detection. The night camera detected 2.47 false positives per hour using a time window designed for our phantom drop tests. However, in real applications this time window can be extended to eliminate false positives caused by nightly active animals. Excluding these from our data reduced the false positive rate to 0.05. The daylight camera detected 0.20 false positives per hour. Our proposed method has the advantages of being more consistent, more objective, less time consuming, and less expensive than manual search methods.

## 1. Introduction

All bat and many wild bird species are protected by EU directives because they are threatened. The bats are all listed in Annex IV and II of the Habitats Directive [1]. This means that member states must undergo steps to maintain, restore and possibly enlarge their natural population [2]. The bird species belonging to Annex 1 of the EU Birds Directive [3] are particularly threatened and it is consequently forbidden to kill or disturb them, so EU member states have to conserve their territories. At the same time, there is significant evidence that birds [4] and bats [5,6] are systematically killed by wind turbines due to the turning rotor. They are either hit or suffer from barotrauma because of the quick pressure changes produced by the rotor blades [7]. This can lead to depletion of their population and, in the worst case, contribute to their extinction. But at the same time, the upcoming climate crisis and our worldwide demand for energy make wind power essential for the future in energy production. Thus mankind has to deal with the conflict between species protection and sustainable energy production. It is also important to keep in mind that the climate crisis itself endangers these species, though in a different manner.

However, there are many attempts to mitigate the collision risk for bats and birds. Wind turbines are turned off at times when the animals are usually active. But that is a drawback for the producers and therefore justifies research for more advanced methods. Some are already in legal use like Probat [8] in Germany, which is a model-based approach for estimating the risk based on sensors. Others are still under investigation like ultrasonic deterrents for bats [9], painted rotor blades for birds [10] and many more. To evaluate the effectiveness of these methods, it is crucial to know the number of individuals being killed. Furthermore, already existing methods may have to be re-evaluated or updated for the use in different countries with different legal situations, species composition, or habitats. In the Black Sea cost region, e.g., the first results on bat fatalities was published in 2020 [11]. Especially with the development of wind farms in new regions which are not covered by existing knowledge, the impacts on wildlife are still under investigation [12] and the number of fatalities is an essential measure. The same holds for research of special species, e.g., Barbastelle bats in Sweden [13]. Also, governments include the searching for carcasses in their guidelines for developing new wind parks, e.g., the Canadian Wildlife Service [14]. Summing it up, there are many investigations going on worldwide concerning the wind power impacts on birds and bats and a majority of them uses the number of fatalities as the main quantification measure.

### State of Research

The state of research method for detecting bat and bird fatalities is a manual search carried out by humans or trained dogs. They search the field in defined spatial patterns and time intervals. These methods are expensive, because they need intensive scheduled visits of the facility. Furthermore, the results vary strongly depending on the search habits of the human or dog, meaning searches are difficult to compare and hardly reproducible. While the detection rate was shown to be better using dogs rather than humans [15], there is still the risk of missing fatalities if the carcasses are carried away by scavengers before the search takes place [16]. According to [17], this effect becomes more obvious with longer search intervals. For these reasons, a 24 h surveillance system would be desirable. One attempt at a technical solution is in the project B-finder [18]. They use rings with thermal cameras filming 360° around the tower in three different heights for detecting falling objects. This requires a minimum of 48 thermal cameras, which leads to an expensive hardware cost. Up to the date of this publication, there are no results known to us. Apart from B-finder, there is no automatic fatality detection system/methodology for birds or bats known to the authors.

## 2. Methods

### 2.1. Basic Concept

Our concept provides a comparably cheap and practicable solution that is easy to implement and able to operate 24/7. The basic idea is a camera system sensing the area below the wind turbine and image processing algorithms analyzing the data. These algorithms compare images at different times around a potential strike event. To properly identify a strike, the contrast between foreground and background has to be sufficient and the images have to be well aligned to each other. Because the nacelle is moving, this presents a challenge and is addressed in detail in Section 2.5. The camera system is based on two different cameras: a visual camera for daytime and a thermal camera for nighttime. As birds are more frequently affected during the day, we use a multispectral VIS–NIR (visual–near infrared) camera, referred to as VIS camera in the following sections. Depending on the background, a filter is used to maximize the contrast. This is shown in Section 2.3.1. Bats are more frequently found around wind turbines between dusk and dawn, so we additionally use a thermal LWIR (long-wave infrared) camera.

The detection algorithms for both cameras have the same basic routines, which will be described together in the following sections. Differences which occur due to the different camera physics (mainly concerning the resolution and the automatic gain control of the thermal camera) will be pointed out explicitly.

**Figure 1:**
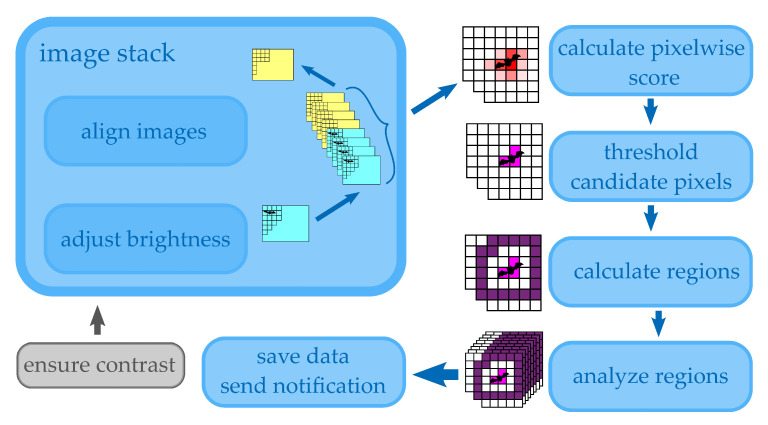
Main steps of developed image processing software (blue) plus contrast as main hardware requirement (gray).

Figure 1 gives a rough overview of the image processing steps that have to be undertaken to get the final result. Firstly, the images are organized in a first-in first-out stack with a fixed number of images per camera. Then, images are aligned to ensure they can be compared on a pixel-by-pixel basis. The last preprocessing step is to adjust the brightness, taking advantage of experimental knowledge about the signal-to-noise ratios. Then, a pixel-wise score is calculated and the pixels are separated by a threshold to find regions with a higher probability of being a potential fatality. These regions are given as input to more specific algorithms where the final decision is made. If the probability is high enough, the data are saved and a notification is sent to do an optional manual search to verify the ground truth. These steps will be outlined as sections below, including an initial section to describe the planned measurement setup.

### 2.2. Planned Measurement Setup

**Figure 2:**
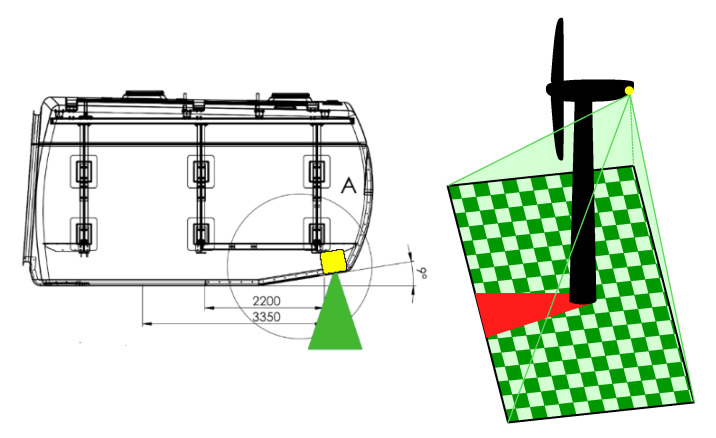
Camera system is mounted on back of nacelle (**left**) and senses area underneath wind turbine (**right**). Tower produces a dead corner which cannot be recorded by system (red projection).

**Figure 3:**
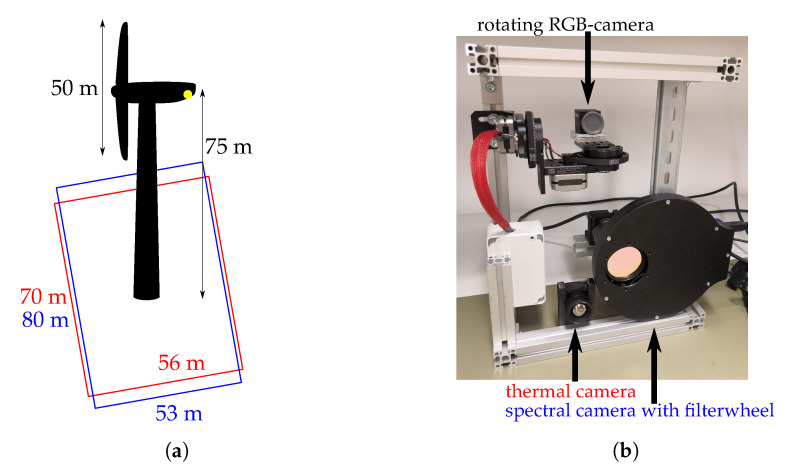
(**a**) dimensions of wind turbine; FOV (= field of view) thermal camera (red); FOV spectral camera (blue). (**b**) Prototype of camera system for collision victim detection.

The system is mounted to the structure itself, at the back side of the nacelle, and facing down to have a good overview of the area below, as shown in Figure 2. The complete system comprises of the two detection cameras introduced in the previous section, a zoom RGB camera optimized for manual control of the detections by humans and a computer for the data processing, as shown in Figure 3. For the camera’s line of sight, it is necessary to fix the aluminum frame in the back side of the nacelle and to drill three holes into its hull. They fit inside an area of 30 cm × 30 cm. The system needs a common AC power supply in the nacelle of about 500 W and an internet connection for notification and remote control. In normal operation mode there is less than 1 GB of memory needed per day, which can be saved in a cloud also. It works in real-time in the sense of doing the fatality detection in parallel of sensing the area. When a collision victim is detected, it raises an event-based alarm and additionally takes a high-resolution zoom picture of that ROI (region of interest). If the collision happens in the darkness, a photo can be taken when the sunlight is present again.

#### Monitored Area and Resolution

Previous research on 39 bat fatalities in Germany showed that 95% are found within a range of about 50 m from wind turbines with a median rotor diameter of 70 m [19]. As the system in our study is designed for a rotor diameter of 50 m, we scaled the values and assume the same relative amount of potential collision victims within 35 m distance. We, therefore, use a thermal camera which is filming an area of 70 m × 56 m and a spectral camera with an area of 80 m × 53 m (Figure 3a). This results in a side length of one pixel projected on the ground of 11 cm for the LWIR camera and about 1.5 cm for the VIS camera. Especially for the LWIR camera, that leads to the necessity of detecting 1 pixel sized objects which makes an extended region analysis of the pixel neighborhood imperative. Note that for a complete estimation of the real fatalities, our detections have to be corrected for the experimentally derived detection sensitivity as well as for the fatalities expected to fall outside of our monitored area.

### 2.3. Contrast

The best possible contrast between background and a potential fatality is crucial for detection reliability. In this section, we describe how we optimized the contrast for both detection cameras. This is described in the following two subsections separately, as they have distinct working principles.

#### 2.3.1. Multispectral Camera

**Figure 4:**
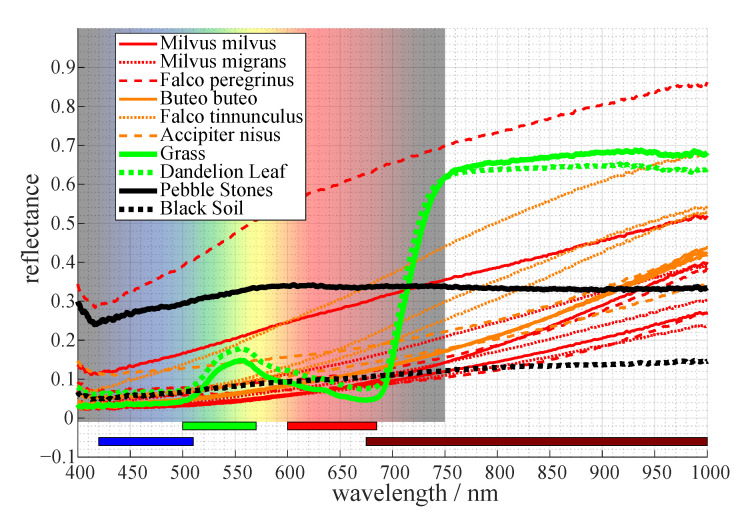
Reflectance of endangered bird species, vegetation, and other possible background surfaces.

The knowledge about the area directly surrounding the wind power plant can be used to maximize the contrast between the background and the potential fatality for the VIS camera. The most common ground of onshore wind turbines consists of grass and other vegetation. The spectrometer measurements in Figure 4 show that healthy vegetation has a minor peak in reflecting light around 550nm. This is the reason why plants are perceived green by humans. However, beyond the visible spectrum, the reflectance increases significantly, beginning at wavelengths of about 700nm.

**Figure 5:**
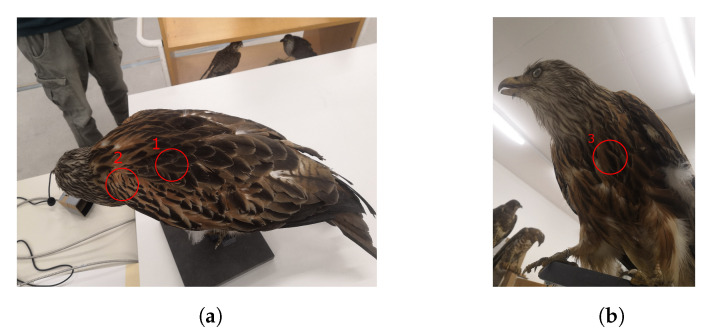
Measurement positions on the back (**a**) and on the front side (**b**) of a Milvus milvus (red kite).

A high reflectance of the background creates the need for a low reflectance of the foreground objects in order to get a reasonable contrast. Medina et al. [20] did spectral measurements on Australian birds. They found that smaller birds in hotter or more arid areas have a higher NIR reflectance. This gives good indication that the reflectance is lower for bigger European birds. To verify this, we did similar measurements of the reflectance of the locally endangered bird species. This was possible due to the archive of the museums of Tyrol which gave us the possibility to use their zoological specimen for our spectrometer measurements. The spectrum of three different parts of their outside feathers was taken, namely below the neck, the side of the main body and the center of their chest, as can be seen in Figure 5.

**Figure 6:**
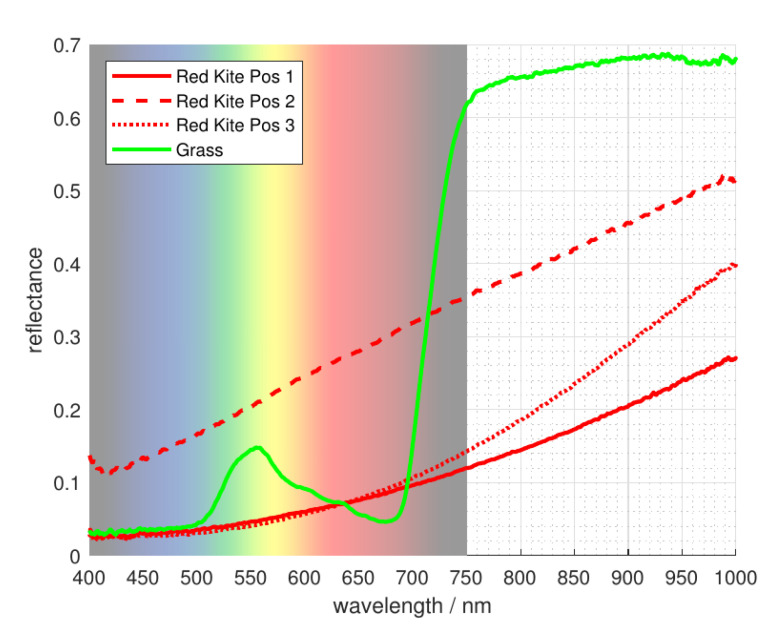
Reflectance measurement result of Milvus milvus (Figure 5) compared to that of grass.

Most of them show the best contrast to grass in the range of about 750nm to about 950nm. This can be seen in Figure 4 and Figure 6, where the reflectance of nearly all bird species (indicated by the red and orange curves) is significantly lower than that of vegetation (indicated by the green curve) in the describes spectral range. The one exception is the measurement of a white feather from the perigrine falcon, indicating our assumption holds for dark feathers, not for light ones. Most endangered birds have mainly dark feathers on the outside for camouflage, so their contrast should be reasonable most times.

Cameras use optical filters to integrate parts of the spectrum over the filter range and save the image as one intergral value per pixel. The color bars on the bottom of Figure 4 indicate the range of different available filters. The brown bar shows a low-pass NIR (near infrared) filter suited to achieve the best contrast for a grass background as described above.

**Figure 7:**
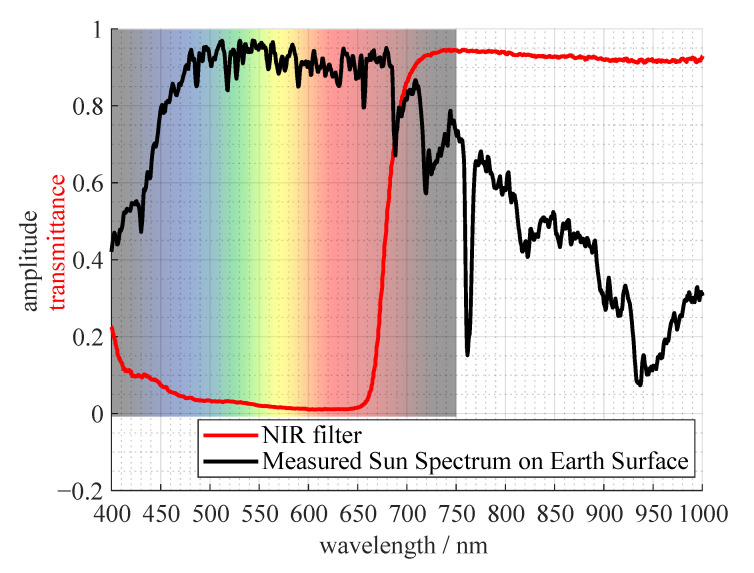
Shape of sun spectrum (normed to maximum of amplitude) measured on earth’s surface; measured transmittance of used NIR filter.

In Figure 7, the transmission curve of the NIR filter is plotted with a representation of the sun spectrum on the earths surface. The spectral energy of the sun is getting lower in the desired region of about 750nm to about 950nm. Together with the low-pass NIR filter it is cutting off the desired region on both sides. The local peak at about 1000nm is irrelevant if all of the hardware is considered, namely the transmittance of the lens and the quantum efficiency of the sensor. The quantum efficiency drops from about 50% at 700nm to below 5% at 1000nm for the Sony IMX183 sensor used in our spectral camera.

Note that if the background has distinct surfaces, other filters may be better suited. For example, for pebble stones, a blue filter could be a good choice. While we planned an automatic filterwheel in our system, we did not use it as the NIR filter best suited our test setup. We use the Flir Blackfly BFS-U3-200S6M-C camera, with the Kowa LM12SC lens and the Baader IR-Pass Filter (685 nm).

#### 2.3.2. LWIR Camera

The contrast of the LWIR camera is physically based on heat radiation and therefore very different to the VIS camera. Microbolometer cameras are reliant on a complex firmware, which automatically compensates for physical effects like drift due to the temperature of the camera itself, the flat field produced by the lens or simply different sensitivities of the pixels. The nonuniformity correction helps to overcome some of these effects. As a next step the measured values get mapped to 8 bit using the Automatic Gain Control (AGC) which contains some adjustable parameters. We increased the linearity parameter which does the mapping in a more linear fashion instead of nonlinearly increasing the detail contrast. While this causes a loss in contrast in the more evenly warmed background, the values more accurately represent the real relative temperatures and result in a better contrast between a fatality and the background. We use the thermal camera Flir Boson 640 with 50° HFOV (Horizontal Field of View) and <40 mK NEDT (Noise Equivalent Temperature Difference).

### 2.4. Image Stack

**Figure 8:**
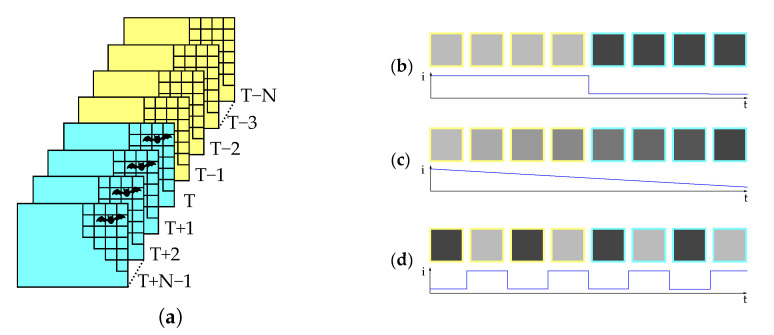
(**a**) Picture stack with new (cyan) and old (yellow) images, (**b**) gray values over time for a potential strike victim, (**c**) change in brightness over time, (**d**) alternating gray values, e.g., moving grass.

For detecting collision victims on the ground, a first-in-first-out image stack is defined as consisting of a fixed amount of images before and after the assumed fatality. To this end, a discrete time rolling window is used with the same number of images before and after the strike. This can be seen in Figure 8 for a variable stack size.

The older half of images in the stack are designated as old and the newer half as new in the following sections. From the computer vision point of view we define a collision victim as an object which is present in all of the new images of the stack, but non of the old ones. This holds for slow frame rates (e.g., 1 measurement per 10 s), because it can be assumed that the object is lying there suddenly. To exclude the risk of catching exactly the moment of the collision victim falling down in one of the images the middle image of the stack may be excluded.

### 2.5. Image Registration Due to Nacelle Movement

To compare the images, it is necessary that they are well registered (image processing terminology for “aligned”). This is usually not the case as the nacelle is changing its direction with the wind and, in addition, there are vibrations present due to the moving parts of the turbine. For the task of finding small objects by comparing images they have to be registered in pixel precision. We use the ORB (Oriented FAST and Rotated BRIEF) [21] open-source algorithm implemented in OpenCV for this task. It is a trade-off between performance and time and for this hardware implementation works quickly enough to **compute the result in the time between two time steps. Additionally, for the registration**, it is crucial that the keypoint detector finds enough unique keypoints in the image. This was never an issue during our phantom drop tests, because the grass and vegetation background offered enough structure.

### 2.6. Image Brightness Adjustment

**Figure 9:**
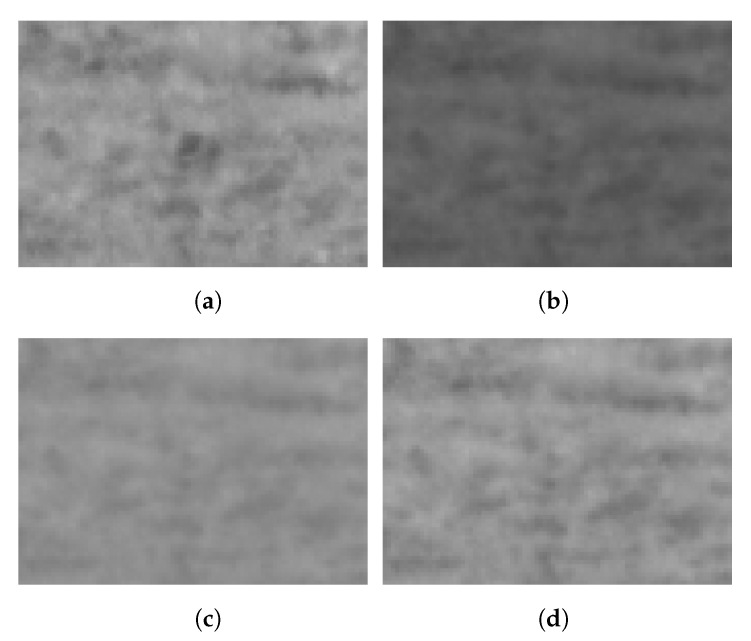
All images show same zoomed part of the scene. In middle of (**a**) phantom can be seen (middle) laying in the grass. One minute earlier (**b**) was taken; object was not there and sun was hidden by clouds. (**c**) is same image as (**b**) but with median adjusted to (**a**). (**d**) is same as (**b**) after histogram matching with respect to (**a**).

Because the compared images are taken with a considerable time delay (e.g., 10 s), the brightness may change due to changing illumination conditions. Clouds may move in front of the sun (VIS camera) and change the brightness of the whole image. This is shown in Figure 9, where (a) was taken about one minute after (b). Furthermore, in the middle of (a), a phantom can be seen. To compensate for the brightness, the median of (b) can be adjusted to (a); this can be seen in (c). The median was taken, as we are interested in a constant brightness of the background over all compared images. Possible fatalities are treated as outliers in this case and do not influence the brightness compensation. Different light conditions additionally result in changes of the contrast. A histogram equalization (d) of all images is able to compensate for the contrast. While this looks like the better compensation to the human eye, the artificial redistribution of the gray values based on another image may means the differences of the absolute values loose their meaning, destroying the detection accuracy of the region analysis. This is why the median is the better choice for compensation and will be applied for the images in the stack before they will be handed over to the pixel-wise detection in the next section.

### 2.7. Pixel-Wise Detection

**Figure 10:**
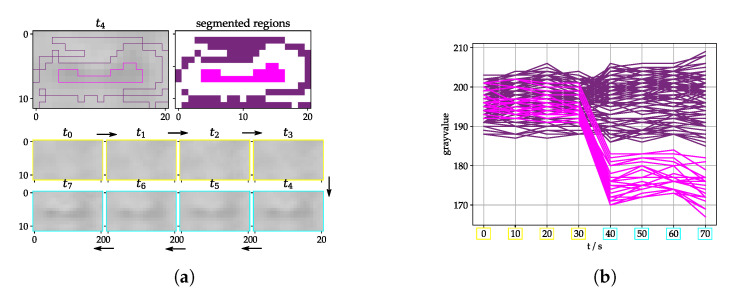
(**a**) Tool for manual segmentation of inner (pink) and outer (purple) region for analysis. $t_{0}$ to $t_{7}$ (newest image) shows image stack according to Figure 8; images are zoomed to relevant part and are with VIS camera (**b**) gray values over time of segmented regions starting from $t_{0}=0$ with Δt=10 s.

To implement a detection algorithm, we need to answer the question: what represents a strike victim from the image processing point of view? To answer this question, we used phantoms with a similar size and texture like real strike victims and tested them outside with a similar background and distance like in the real setup. The images were analyzed with a manual segmentation tool which was built for that reason. The tool offers the possibility to look at the images over time in form of a stack (Section 2.4), segment regions and plot the curves of the pixel gray values over time (Figure 10).

**Figure 11:**
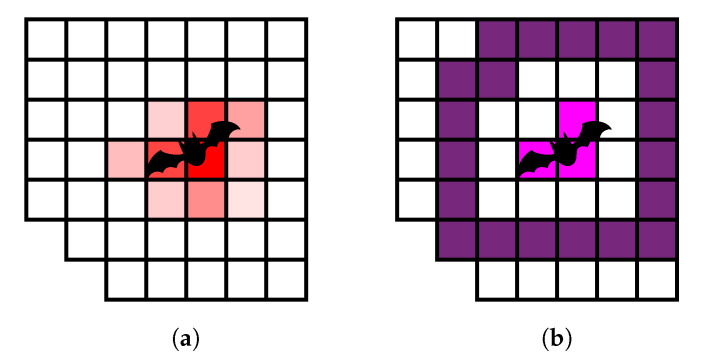
(**a**) Fatality score (red) and ground truth; (**b**) resulting region after thresholding and morphological operations.

There was no strike victim from $t_{0}$ to $t_{3}$, but a dark object is lying in the grass from $t_{4}$ to $t_{7}$. So the inner pixels (pink) change their value from $t_{3}$ to $t_{4}$, but the outer pixels (purple) do not. Therefore, the first tactic is to calculate a pixel score to find those with a relevant step from $t_{3}$ to $t_{4}$ but just small changes between the other time steps. Pixel-wise methods have the advantage of a constant time consumption for each frame and they are usually not very computationally expensive compared to methods with inter-pixel dependencies. The method is used for selecting pixels with a higher probability of being part of a fatality, mentioned as score in the following sections (Figure 11a).

The ideally expected sequence of pixel gray values as shown in Figure 8b, showing a sudden transition from a constant high gray value to a constant low gray value. The gray values can also change due to external reasons like different illumination by sunlight and the resulting change of exposure time and gain of the camera. This functionality is necessary to stay within the detectable range. However, these changes are comparably slow and should either be small enough or happen over more than one image (Figure 8c). Other unwanted signals arise through moving objects like grass in the wind or animals walking through the scene (Figure 8d). These signals should not lead to a high detection score (Equation (1)).

The score for the VIS camera is calculated by

(1) $$S_{\mathrm{VIS}}=-\mathrm{LUTshift}\left(I_{T}-I_{T-1}\right)-\frac{\alpha }{N-1}\times \sum_{t=0}^{N-2}|I_{T+t}-I_{T+t+1}|-\frac{\alpha }{N-1}\times \sum_{t=-2}^{-N}\left|I_{T+t}-I_{T+t+1}\right|$$

where S is a score matrix, I is the image matrix with the time step as index, *N* is the half size of the picture stack, α is a fixed weighting factor and LUTshift is function to change the gray value distribution.

**Figure 12:**
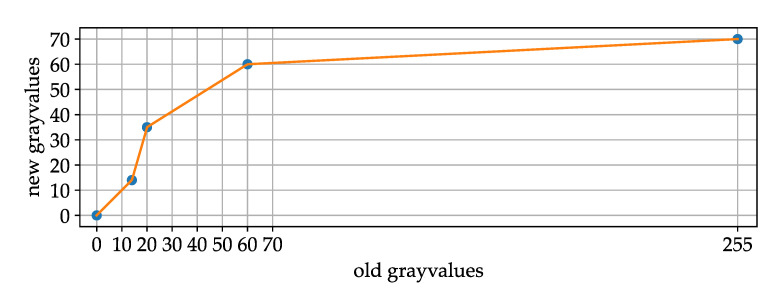
LUT for LUTshift function in Equation (1).

Test data made clear that it makes sense to adjust the image gray value distribution for the difference image in the first part of Equation (1). We called this function LUTshift as it is basically a LUT (lookup table). By manually analyzing test data, it can be seen that the difference representing an artificial collision victim is often in the range from about 15 to 30. Lower values are more often a result of noise. Values above 70 are very rarely fatalities so it helps to lower their score to not overestimate them. Figure 12 shows that a maximal value of 255 maps to 70.

**Figure 13:**
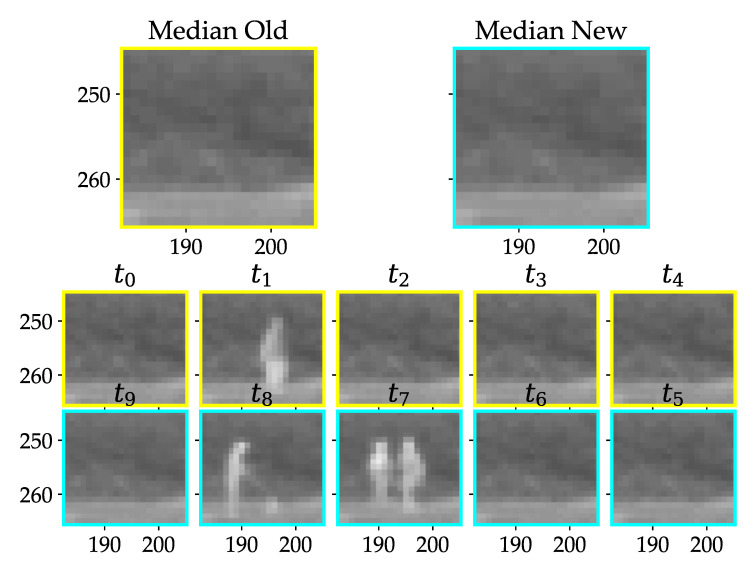
Moving people recorded with the LWIR camera; the median helps to get rid of outliers.

To calculate the pixel score for the LWIR camera, the median over time is taken for the old (Section 2.4) and for the new images. The score is their difference. The median helps to get rid of outliers like an animal moving through the scene (Figure 13).

(2) $$S_{\mathrm{LWIR}}=\mathrm{median}\left(I_{T},...,I_{T+N-1}\right)-\mathrm{median}\left(I_{T-1},...,I_{T-N}\right)$$

### 2.8. Region Generation

**Figure 14:**
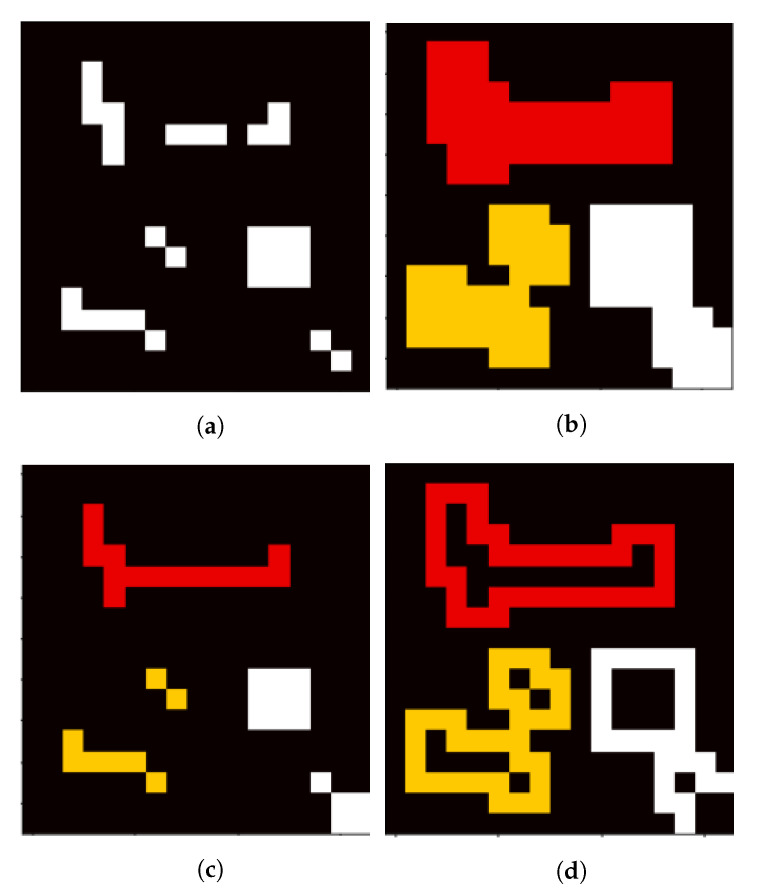
Region Generation: (**a**) thresholded image, (**b**) labeled *full*, (**c**) labeled *inner* and (**d**) labeled *outer* areas.

To separate high from low scores, adaptive thresholding [22] is used. This method helps to find local maxima instead of using a global threshold which is not always appropriate e.g., when a part of the image is in the shadow or the grass is not uniformly heated (for the LWIR camera). This produces a binary map like in Figure 14a. The thresholded pixels are on the one hand not necessarily representing the whole strike victim and on the other hand are not providing information about the surrounding area. Therefore, it is necessary to define regions based on the pixels for further analysis. As a pre-processing step morphological opening can be applied to get rid of very small pixel clusters. Then dilation is applied to connect very close pixels and also include the surrounding of the potential strike victim (Figure 14b). In this state, a connected components analysis is done to derive distinct regions, allegorized in different colors here. By erosion and subtraction it is possible to further split the regions into the inner, outer and full region (Figure 14b–d respectively). The described order of the morphological operations and the labelling is crucial to receive an equal number of inner, outer, and full regions, because morphological operations are able to change the number of connected components in general.

After this step, it is possible to iterate over the regions and do further analysis on them. Figure 11b shows an example of one region with inner and outer area. It can be seen, that the inner region is assumed to be the strike victim and the outer region its surrounding area. The space in between can be adjusted by the number of morphological operations and exists to clearly separate inner and outer region for further investigation. The *full* region adds up the inner and outer region and the space in between.

### 2.9. Region Analysis

The parameters of the analysis task are significantly different for both cameras. This is mainly due to the fact that the resolution is very different. Additionally, the LWIR camera uses AGC algorithms to compensate for physical camera effects and has a different SNR also.

Due to the fact that the objects are very small, consisting of about 1 to 20 pixels, it is not possible to use usual object detection algorithms relying on morphological or texture properties. [23] describe a similar problem detecting honey bees with drones. But their objects are consisting of about 100 pixels and they have enough labeled test data to use convolutional neural networks for the background subtracted images. Our objects are smaller, but we use the fact that they will most likely not move after the strike, making the detection task very specific. Another example is given in [24], where they detect small low contrast airplanes. They use a Kalman filter to estimate the movement which is appropriate for their task but not for ours, showing again that specific solutions are needed for such specific problems.

**Figure 15:**
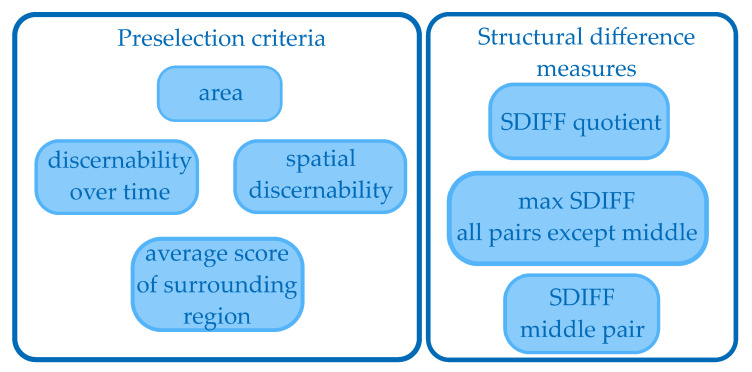
Criteria for the decision about a detection.

After analyzing the test videos we identified the criteria and their thresholds crucial to decide if a collision victim is detected or not. The criteria can be seen in Figure 15, where the left side shows the preselection criteria described in Section 2.9.1 and the right side indicates the Structural Difference (=SDIFF) measures described in Section 2.9.2.

**Figure 16:**
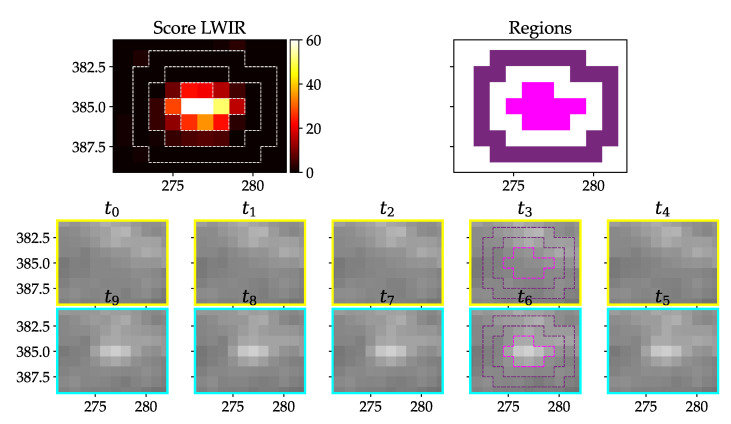
Warm test object (body temperature) with about 5 cm lengths lying in grass; upper left: resulting score from pixel-wise detection; upper right: derived inner (pink) and outer (purple) region; two lower rows: image stack from oldest ($t_{0}$) to newest ($t_{9}$) image with assumed strike happening between $t_{4}$ and $t_{5}$.

In Figure 16, an example of a true positive phantom detection with the LWIR camera can be seen. All frames show the same zoomed part of the image.

#### 2.9.1. Preselection

The preselection criteria are those which are easy and quick to compute. The aim is to remove the most unrealistic candidates and perform the more computationally expensive work on just the remaining ones. The area gives information about the size of the object and is therefore a good measure for preselection. All criteria thresholds have to be adjusted differently for LWIR and for VIS images.

Additional measures for preselection are the discernibility over time and space by their gray value and the average score of the surrounding region. For the LWIR camera we assume the strike victim to be warmer than its surrounding. This would mean that the inner pixels are both significantly warmer than the outer pixels and warmer than the full region of the old images. The definition of the criteria is listed in Table 1.

The result for the example in Figure 16 is shown in Table 2. All of the values are within the defined ranges.

**Figure 17:**
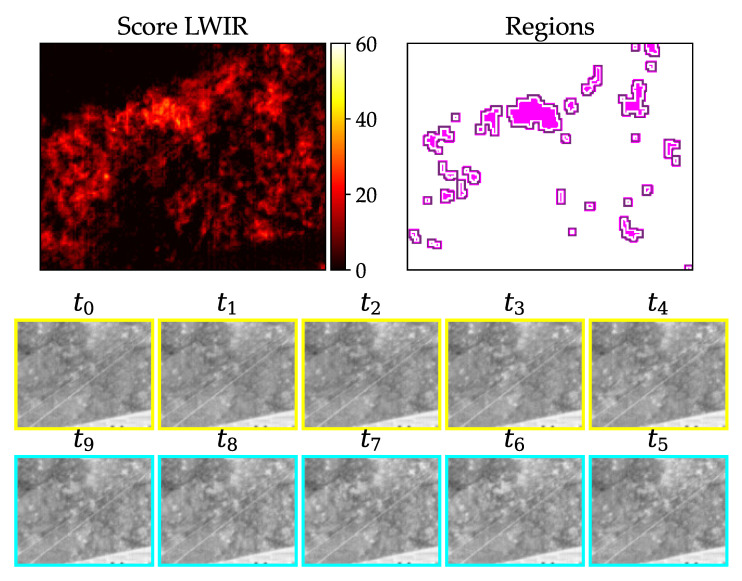
Wind leads to a high LWIR score distributed over a big area compared to a potential detection.

Test videos show that there is a dependency between the measured values and wind gusts, because the angle of the grass influences the main direction of heat radiation of the grass surface (LWIR camera). An example can be seen in Figure 17, where the average LWIR score around the single regions is unusually high compared to a true positive detection. We therefore neglect detections if the average score within a certain distance is above a defined threshold.

#### 2.9.2. Structural Analysis

**Figure 18:**
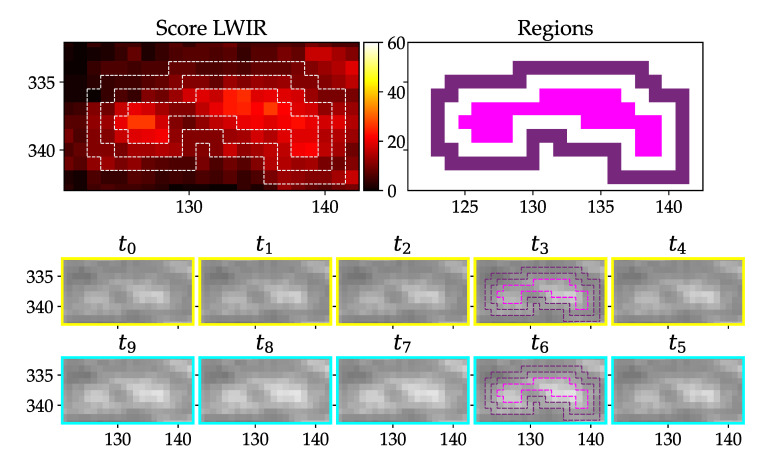
Grass getting illuminated by sun differently over time, which leads to a false positive detection after preselection, but obviously image content stays same from $t_{4}$ to $t_{5}$.

After the preselection process, there are many false positives left and it is crucial to further reduce them. One cause for false positives (LWIR camera) is different directional absorption and emissivity of materials, which can be seen in Figure 18, where parts of the grass is unequally heated by the sun. The image content over time looks very similar to the human eye, but the LWIR Score is still significant. So another measure has to be used which concentrates more on the interpixel structure.

**Figure 19:**
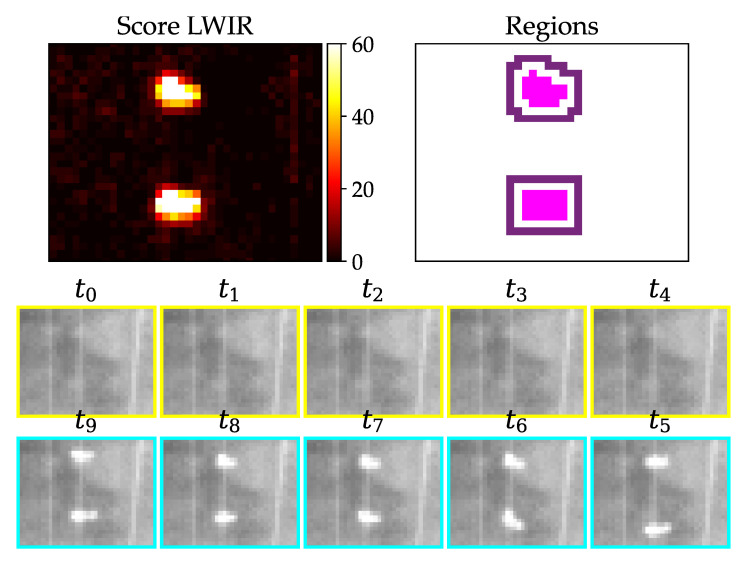
Birds sitting on a guy wire produce false positives without further filtering.

Another kind of false positive arises due to slightly moving objects like birds sitting in the grass or on a cable. An example can be seen in Figure 19. Grass, which is moving due to wind, can show similar effects.

From a human perspective, it is obvious that these two false positives can be dismissed by finding no significant change in content from $t_{4}$ to $t_{5}$ (Figure 18) or too much content change in the rest of the images (Figure 19). So we need a measure which accounts more for the structural differences between the images. Thus, the effects of absolute illumination change can be considerably diminished or movement can be detected. The measure needs the ability to be computed on nonsquare regions and has a preferred time complexity of no higher than O(n). The Structural Similarity (=SSIM) from [25] was tested and turned out to be suitable for that requirements. It is usually used to rate the human perceived quality of a reference image x compared to a compressed version y of the same image. It uses only statistical measures like mean, variance and covariance for calculation and is therefore computationally less expensive than algorithms with a higher complexity, e.g., sorting algorithms. We use the SSIM two compare all pixels of one full region between two consecutive time steps. We use the usual form, where α=β=γ=1:

(3) $$\mathrm{SSIM}\left(x,y\right)=\frac{\left(2\mu_{x}\mu_{y}+C_{1}\right)\left(2\sigma_{xy}+C_{2}\right)}{\left(\mu_{x}^{2}+\mu_{y}^{2}+C_{1}\right)\left(\sigma_{x}^{2}+\sigma_{y}^{2}+C_{2}\right)}$$

In the above equation, $\mu_{x}$ and $\mu_{y}$ are the mean values, $\sigma_{x}$ and $\sigma_{y}$ are the variances and $\sigma_{xy}$ is the covariance of the region. The SSIM takes values between 0 (=no similarity) and 1 (=total similarity).

**Figure 20:**
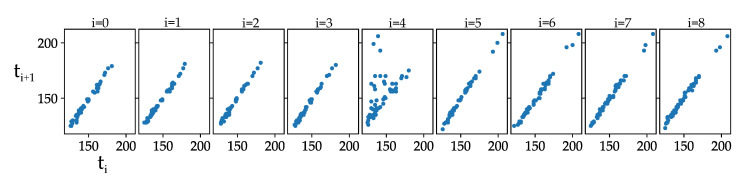
Gray value vector of region at time $t_{i}$ on x-axis plotted against vector of the same region at time $t_{i+1}$ on y-axis of true positive detection (Figure 16).

In Figure 20, the gray value vectors of consecutive time steps are plotted against each other. The resulting SSIM can be seen in Table 3. The plots belong to Figure 16 (true positive detection). If two of the same images would be plotted on the x-axis and on the y-axis, that would result in a straight 45° alignment of points. A high SSIM correlates to values which are in a linear fashion. In the middle time step, they have no clear linear equivalent and the SSIM is lower.

**Figure 21:**
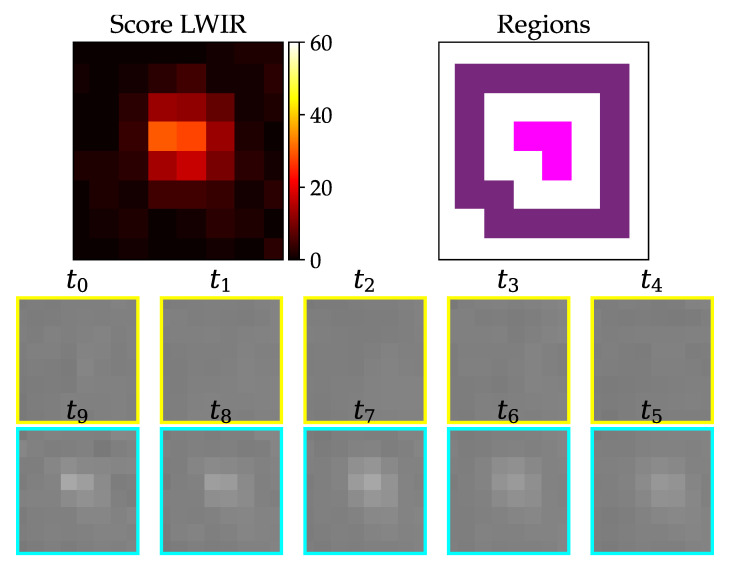
Phantom with similar gray values as background.

**Figure 22:**
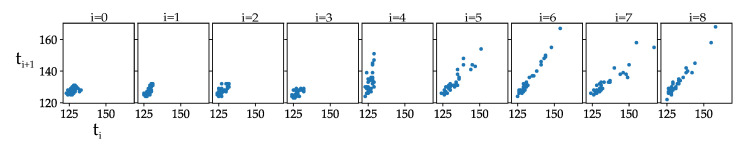
Gray values of vector $t_{i}$ on x-axis and vector $t_{i+1}$ on y-axis for image data of Figure 21.

$C_{1}$ and $C_{2}$ (Equation (3)) are described to stabilize the denominator, if it is close to zero. Concerning our patches, $C_{2}$ helps to derive a higher similarity between patches which have very close gray values (low variances), and thus their noise would otherwise dominate the calculation. This is relevant for Figure 21, because the absence of the object leads to very uniform gray values. The concerning plots are shown in Figure 22. The SSIM of image pairs from $t_{0}$ to $t_{4}$ is very dependent on $C_{2}$ as can be seen in Table 4.

We use a $C_{2}$ of 0.03 (Table 4) which is a good value to compensate for the noise (image pairs 0–1 to 3–4) and result in relatively high values compared to the middle similarity (image pair 4–5), which results in a significantly lower similarity. This characterizes a successful detection. To make it more convenient for calculations, we define the Structural Difference as

**Figure 23:**
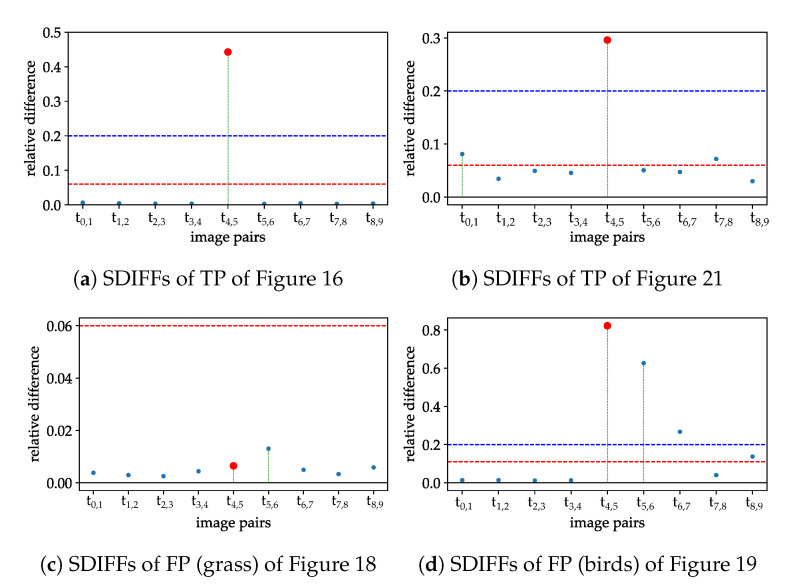
SDIFFs with middle SDIFF in red and the maximum of outer SDIFFs in blue with green distance line. Horizontal lines are the thresholds according to Table 5. (**a**,**b**) are true positives, (**c**,**d**) are false positives from preselection. (**c**,**d**) are correctly dismissed through SDIFF criteria in Table 5.

(4) SDIFF=1−SSIM

because we expect small differences between all image pairs except the middle pair, and want to be sensitive to that. The SDIFFs of the true and false positives from above are shown for comparison in Figure 23.

Figure 23 shows the performance of the SDIFF measure for different LWIR examples. The red dot is the SDIFF of the middle image pair and the red line is the threshold described in Table 5. (a) and (b) are finally labeled as true positives, because the red dot is greater than the threshold, all blue dots are smaller and the quotient between the red and the maximum blue dot is greater than the threshold of criterium 6. (c) gets dismissed due to the middle SDIFF being too low because the middle image pair is too uniform and, in addition, the quotient is too small. (d) is the representation of the lower birds SDIFFs. This example is dismissed, because the the bird is not sitting still, which can be seen especially from $t_{5}$ to $t_{6}$.

If the points are somewhere between the two thresholds from criteria 4 and 5, the SDIFF quotient helps to make the final decision if the SDIFF of the middle time step is relatively more elevated than all of the other ones.

### 2.10. Test Series of Prototype

**Figure 24:**
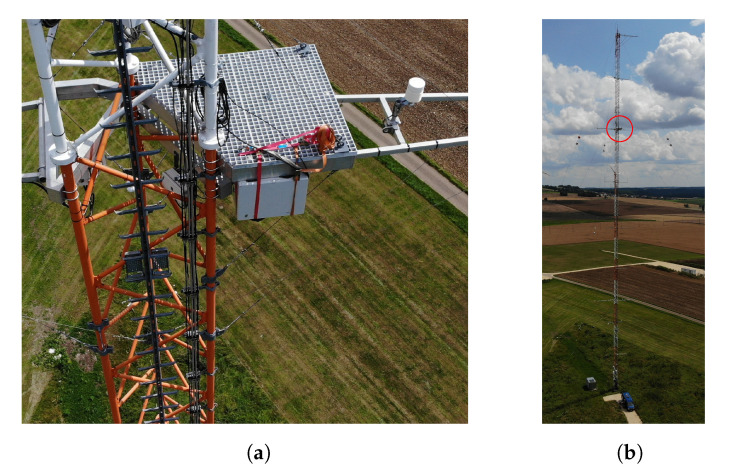
The camera system in the gray box (**a**) was mounted in 75 m height (**b**).

The planned wind turbine has not yet been installed, therefore we tested our system on a met mast in the same field on the Kuchalb in Germany just 130 m away from the actual place. The computer vision system was installed at a height of 75 m facing downwards (Figure 24). We had partly sunny and cloudy weather conditions with wind gusts up 16 m per second on the ground.

**Figure 25:**
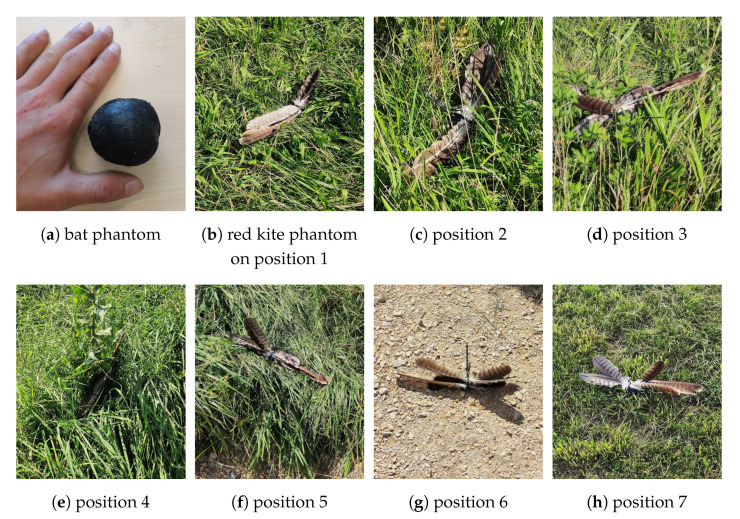
The bat phantom for the LWIR camera (**a**) and the red kite phantom for the spectral camera (**b**–**h**) were placed on different defined positions including grass with various lengths (**b**–**f**,**h**) and gravel road (**g**).

We built animal phantoms to simulate collisions (Figure 25). For the bat detection, we used a black balloon filled with sand and water and heated it up to a temperature between 32 °C and 36 °C, before dropping it on defined positions. For the detection of birds we used some contour feathers from a red kite (Milvus milvus) and tied them together.

**Figure 26:**
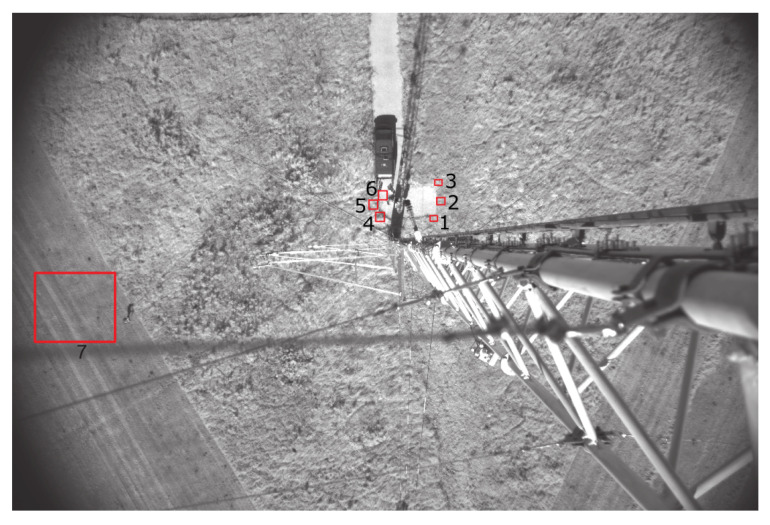
Positions for phantom drops (VIS and LWIR).

The phantoms were placed on defined positions including different grass lengths and gravel road. For later analysis, the time stamps were logged. In Figure 26 the absolute positions can be seen in a VIS images taken from the camera system.

## 3. Results

### 3.1. LWIR Results

We laid out phantoms at the 11th of August 2021 from 9:23 p.m. local time to 9:53 p.m. and on the 12th from 6:33 a.m. to 7:07 a.m.

**Figure 27:**
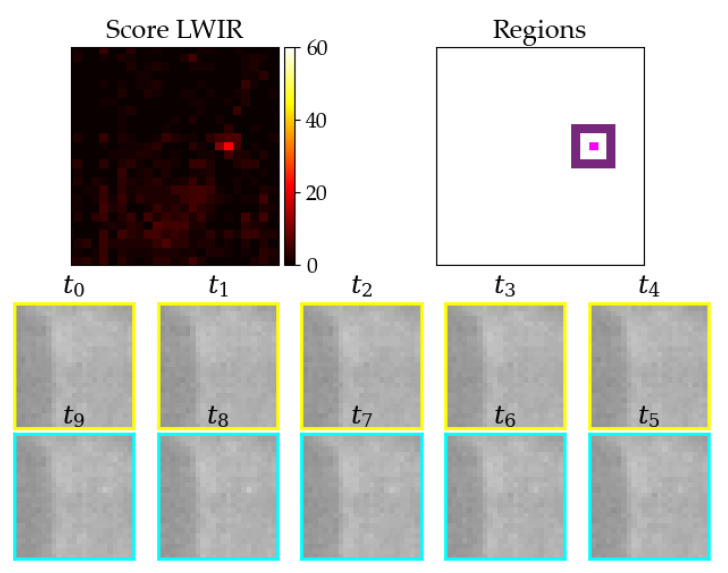
Example detection of balloon phantom at about 35 °C in middle high grass at position 2.

We did 41 drops on positions 1, 2 and 6 (Table 6). Positions 1 and 2 were grass with heights of about 10 to 50 cm which were representative for most of the field. An example detection can be seen in Figure 27. Position 6 was gravel road. We did no drops on positions 5 and 6 (grass height of about 80 cm), where the balloon would were hidden completely, making detections essentially impossible. True positives and false negatives (FN) are produced by the drop tests and false positives are defined as unexpected detections.

There were no false positives during the drops. Measurements were done also in the night from the 10th to the 11th of September and from the 11th to the 12th. The results are summarized in Table 7. Civil twilight times on the 11th were from 5:33 a.m. to 6:09 a.m. and from 8:47 p.m. to 9:22 p.m. according to [26].

That makes a rate of 2.47 false positives per hour where most of them are produced by night active animals. This will be discussed in more detail in Section 4.

The birds sitting on the cable in the morning can be neglected, because on an actual wind turbine, there is no cable. Although interpretation of 1 pixel events is difficult, the majority of false positives were likely produced by animals in the field, as movement could be seen in the temporal and spatial proximity to these detections.

**Figure 28:**
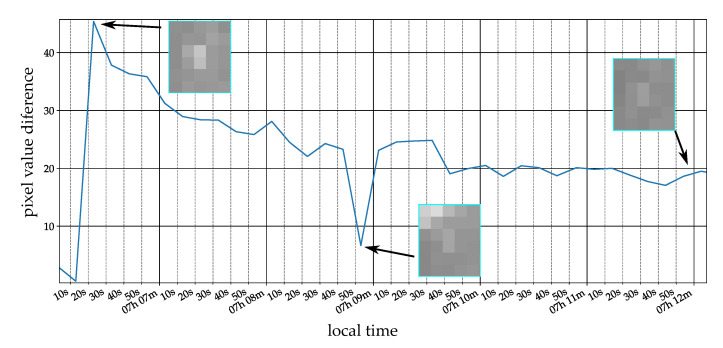
Two pixel bat phantom cooling down over time.

However, to overcome this issue in the future we analyzed the time for the phantom to cool down, which can be seen in Figure 28 for a two pixel phantom. The curve represents the difference between the mean of the inner and outer region. The curve looks similar to newtons law of cooling and that there is a detectable heat radiation also about 5 min after dropping the object. According to this result, it would be possible to enlarge the time frame for our image stack to reduce false positives due to nightly active animals, see Section 4 for more details.

### 3.2. VIS Results

**Figure 29:**
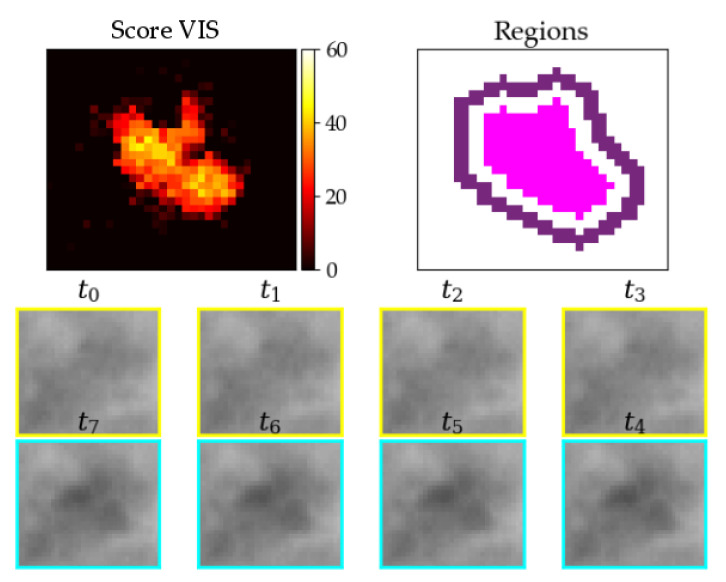
Example detection of red kite phantom at about 35 °C in middle high grass at position 2.

We laid out phantoms on the 11th of August 2021 from 10:40 a.m. local time to 12:00, from 5:53 p.m. to 6:15 p.m., and from 7:16 p.m. to 7:55 p.m. We did 108 drops on positions 1, 2, 3, 4, 5, 6, and 7 (Table 8). An example detection on position 2 can be seen in Figure 29.

**Figure 30:**
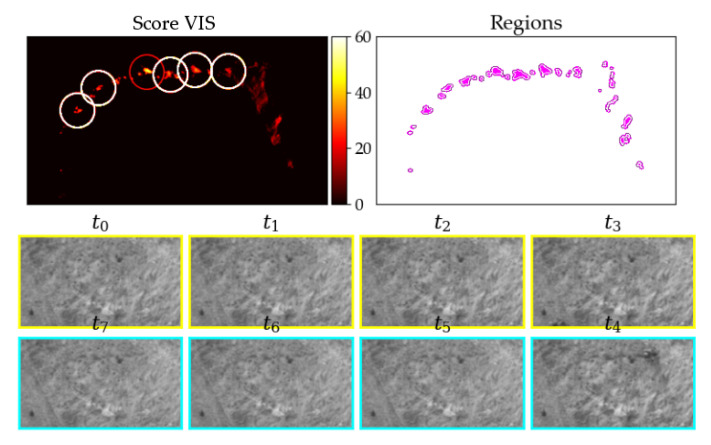
Footsteps in high grass can produce false positives.

We missed some of the phantoms due to the high grass on positions 4 and 5, when the phantom got hidden in the high grass. On positions 6 and 7 the problems were mainly difficult light conditions. Either the light was changing over time due to moving clouds or the light was too low. The overall sensitivity was 84.3%. During our measurements we produced 6 false positives. Three of them were produced by our chair, 2 by our steps in the grass, which produce a shadow in the high grass (Figure 30) and one from the traces in the grass of a mowing tractor.

To quantify the false positive detection rate we did measurements at the times listed in Table 9. This makes a total time of 10 h 5 min. The false positives were either produced by wind, shadow, a tractor or ourselves. Latter two can be neglected due to their artificial origin leaving two false positives. This makes a rate of 0.20 relevant false positives per hour.

## 4. Discussion

An uncertainty of our design is the way real bats and birds die after a collision with the wind turbine. Our concept provides for the idea that they remain motionless after hitting the ground, which may not be the case, if they are still alive. Grodsky et al. [27] states that there are indications that a significant share of the bats do not die immediately after getting hit. This obviously leads to wrong numbers, but also manual search is just an extrapolation for the real number of fatalities. Our method has to be evaluated by manual search to make it comparable. This will be carried out in the progress of the project.

It is difficult to get enough ground truth data for this issue, because just about 2 to 10 dead bats are expected over one year and also bird collisions are seldom events. However, we still hope to obtain more information on this when the test turbines are built and we are able to install our system.

A further uncertainty is the exact effect of the nacelle vibrations on the registration accuracy of the ORB algorithm. Due to a lack of the ground truth, it is difficult to test. On our detected phantom drops, the registration at the phantom was always in pixel precision, but we had not much displacement and vibration on the met mast. It is necessary to reevaluate that when the system is finally mounted in the nacelle. We did a few small trials of shaking the frame of our system while filming. The outcomes suggest that the affine registration works in pixel precision as long as there are enough keypoints in the images. The thermal camera images as well as the spectral camera images with the grass background were rich on keypoints, so we believe more vibration will be no problem.

We did our drop tests mainly on two days at the test side. It was partly cloudy and there was wind blowing from about 0 to 16 m/s. The system has to be tested in more diverse weather conditions to get a more reliable dataset. Nevertheless, the windy conditions gave us already the possibility to understand more about the effects of wind and high grass on the image data.

### 4.1. False Positive Detection of Nightly Active Animals during the LWIR Measurements

By analyzing the results of the LWIR measurements, it became clear that nightly active animals are sitting still long enough to produce false positives if the monitored time window is too short. In our test setup that time was 50 s and we detected 48 living animals in two nights. Investigation of these false positives show that it is possible to considerably reduce them by extending the time window by about factor 4. The cooling curve experiments indicate that this will not significantly lower the sensitivity on the true positives. Excluding the nightly active animals, the false positive rate in our phantom drop tests would get reduced from 2.47 per hour to 0.05 per hour. Another possibility to exclude these FP is to use a higher frame rate for the image stack and filter them by detecting their movement on the ground.

### 4.2. Comparison of Our Proposed Method to Manual Search Methods

The sensitivity of the nighttime detection can be partly compared to studies about human and dog manual search of bat carcasses. In our study, we derived sensitivities between 71.4% (grass) and 80.0% (open space). Brinkmann et al. [28] documented a human search efficiency of 75% in open space and 66% in high grass (without quantitatively defining the grass height). Smallwood et al. [15] report about a human search efficiency of 6% and 96% for dogs, Mathews et al. [29] of 20% for humans and 73% for dogs, Arnett et al. [30] of 14%/42% for humans and of 71%/81% for dogs and Dominguez et al. [31] of 20% for humans and 80% for dogs. From this we can infer that the resulting efficiency is dependent on many factors and very inconsistent among different studies. A disadvantage of manual search is the removal rate of dead animals by scavengers. Brinkmann et al. [28] investigated the amount of dead animals not getting carried away by scavengers in the first 24 h. The value was 79% on average, but had a high variation between 47% and 90% on different facilities. Assessing the correct removal rate is a complex task and is also dependent on many factors. In comparison to manual search methods, our suggested method has the disadvantage of producing some false positives every night. But it is possible to manually sort them out in front of the computer without the necessity of visiting the facility and searching for the object. Additionally, we believe that it is possible to further reduce the false positives with our new data generated from the phantom drop test.

In summary, our method provides a similar sensitivity as manual search methods but produces a more unbiased data set, operates in real-time, and makes the results on different wind turbines more comparable.

## Figures and Tables

**Table 1:** Definition of the preselection criteria.

#	Criterium Name	Criterium Rule
1	area	within limits
2	discernability over time	median(new, inner) − median(old, full) > threshold 1
3	spatial discernability	median(new, inner) − median(new, outer) > threshold 2
4	average score in surr. region	<threshold 3

**Table 2:** Preselection criteria for test object in Figure 16.

#	Criterium for LWIR Image	Calculated Value
1	≥1 and ≤100	10
2	>11	27
3	>11	36
4	<2.9	0.24

**Table 3:** SSIM in % for true positive detection (Figure 20).

t	0–1	1–2	2–3	3–4	4–5	5–6	6–7	7–8	8–9
	99.3%	99.6%	99.6%	99.7%	55.7%	99.7%	99.5%	99.7%	99.6%

**Table 4:** SSIM in % for vector plot in Figure 22.

t	0–1	1–2	2–3	3–4	4–5	5–6	6–7	7–8	8–9
$C_{2}$ = 0.0	34.9%	71.8%	65.8%	61.7%	31.4%	91.6%	93.3%	90.1%	95.8%
$C_{2}$ = 0.01	63.4%	84.3%	79.4%	79.0%	40.1%	92.2%	93.6%	90.5%	96.0%
$C_{2}$ = 0.03	91.9%	96.6%	95.1%	95.4%	70.4%	94.9%	95.3%	92.8%	97.0%

**Table 5:** Definition of structural difference criteria.

#	Criterium Name	Criterium Rule
4	SDIFF of middle image pair	>threshold 3
5	max SDIFF of side image pairs	<threshold 4
6	SDIFF quotient	$\frac{\mathrm{crit}.\;4}{\mathrm{crit}.\;5}$ > threshold 5

**Table 6:** Results of bat phantom drop tests.

Position	Duration	TP	FN	FP	Sensitivity
1 & 2		15	6		71.4%
6		16	4		80.0%
all	1 h 4 min	31	10	0	75.6%

**Table 7:** False positives during nights from 10th to 12th.

Night	Duration	FP	Reason
10th 9:04 p.m.–11th 7:30 a.m.	10 h 26 min	28	1 to 3 pixel animal in field
		4	bird sitting on guy cable
11th 8:30 p.m.–12th 7:30 a.m.	11 h	20	1 to 3 pixel animal in field
		1	wind changing radiation direction

**Table 8:** Results of red kite phantom drop tests.

Position	Duration	TP	FN	FP	Sensitivity
1		14	1		93.3%
2		16	0		100.0%
3		10	0		100.0%
4		14	6		70.0%
5		8	2		80.0%
6		14	3		82.3%
7		15	5		75.0%
all	2 h 21 min	91	17	6	84.3%

**Table 9:** VIS False positives on 11th of August.

Time	Duration	FP	Reason
9:41 a.m. to 1:41 p.m.	4 h	2	traces in the grass (people or tractor)
		9	repositioning of non-phantom objects by ourselves
		1	wind
2:39 p.m. to 5:50 p.m.	3 h 11 min	2	repositioning of non-phantom objects by ourselves
5:53 p.m. to 8:47 p.m.	2 h 54 min	6	traces in the grass (people)
		10	repositioning of non-phantom objects by ourselves
		1	shadow of the metmast

## Data Availability

Data of the drop tests and spectral curves of the mentioned bird species can be sourced from the corresponding author, C.H.

## Abbreviations

The following abbreviations are used in this manuscript:

AGC	Automatic gain control
BRIEF	Binary Robust Independent Elementary Features
FAST	Features from Accelerated Segment Test
FN	False negatives
FOV	Field of view
FP	False positives
HFOV	Horizontal Field of View
LWIR	Long wave infrared
NEDT	Noise Equivalent Temperature Difference
NIR	Near infrared light spectrum
ORB	Oriented FAST and Rotated BRIEF
ROI	Region of interest
SDIFF	Structural difference
SNR	Signal to noise ratio
SSIM	Structural similarity
TP	True positives
VIS	Visible light spectrum

## References

[1] EU Habitats Directive. https://ec.europa.eu/environment/nature/legislation/habitatsdirective/index_en.htm.

[2] Rodrigues L., Bach L., Dubourg-Savage M.J., Karapandža B., Rnjak D., Kervyn T., Dekker J., Kepel A., Bach P., Collins J. (2015). Guidelines for Consideration of Bats in Wind Farm Projects Revision 2014.

[3] Threatened Bird Species in Annex, I. https://ec.europa.eu/environment/nature/conservation/wildbirds/threatened/index_en.htm.

[4] Reichenbach M., Steinborn H. (2006). Windkraft, Vögel, Lebensräume–Ergebnisse einer fünfjährigen BACI-Studie zum Einfluss von Windkraft-anlagen und Habitatparametern auf Wiesenvögel. Osnabrücker Naturwissenschaftliche Mitteilungen.

[5] Arnett E.B., Brown W.K., Erickson W.P., Fiedler J.K., Hamilton B.L., Henry T.H., Jain A., Johnson G.D., Kerns J., Koford R.R. (2008). Patterns of bat fatalities at wind energy facilities in North America. J. Wildl. Manag..

[6] Baerwald E., Patterson W., Barclay R. (2014). Origins and migratory patterns of bats killed by wind turbines in southern Alberta: Evidence from stable isotopes. Ecosphere.

[7] Baerwald E.F., D’Amours G.H., Klug B.J., Barclay R.M. (2008). Barotrauma is a significant cause of bat fatalities at wind turbines. Curr. Biol..

[8] Behr O., Brinkmann R., Hochradel K., Mages J., Korner-Nievergelt F., Niermann I., Reich M., Simon R., Weber N., Nagy M. (2017). Mitigating bat mortality with turbine-specific curtailment algorithms: A model based approach. Wind Energy and Wildlife Interactions.

[9] Weaver S.P., Hein C.D., Simpson T.R., Evans J.W., Castro-Arellano I. (2020). Ultrasonic acoustic deterrents significantly reduce bat fatalities at wind turbines. Glob. Ecol. Conserv..

[10] May R., Nygård T., Falkdalen U., Åström J., Hamre Ø., Stokke B.G. (2020). Paint it black: Efficacy of increased wind turbine rotor blade visibility to reduce avian fatalities. Ecol. Evol..

[11] Măntoiu D.Ş., Kravchenko K., Lehnert L.S., Vlaschenko A., Măntoiu D.Ş., Kravchenko K., Lehnert L.S., Vlaschenko A., Moldovan O.T., Mirea I.C. (2020). Wildlife and infrastructure: Impact of wind turbines on bats in the Black Sea coast region. Eur. J. Wildl. Res..

[12] Aschwanden J., Stark H., Peter D., Steuri T., Schmid B., Liechti F. (2018). Bird collisions at wind turbines in a mountainous area related to bird movement intensities measured by radar. Biol. Conserv..

[13] Apoznański G., Sánchez-Navarro S., Kokurewicz T., Pettersson S., Rydell J. (2018). Barbastelle bats in a wind farm: Are they at risk?. Eur. J. Wildl. Res..

[14] Canadian Wildlife Service (2007). Wind Turbines and Birds: A Guidance Document for Environmental Assessment. Canadian Wildlife Service.

[15] Smallwood K.S., Bell D.A., Standish S. (2020). Dogs Detect Larger Wind Energy Effects on Bats and Birds. J. Wildl. Manag..

[16] Parisé J., Walker T.R. (2017). Industrial wind turbine post-construction bird and bat monitoring: A policy framework for Canada. J. Environ. Manag..

[17] Smallwood K.S. (2017). Long search intervals underestimate bird and bat fatalities caused by wind turbines. Wildl. Soc. Bull..

[18] B-Finder System, 24 Month Test Report for T-Series. https://b-finder.eu/publication/B-FINDER_general_DE.pdf.

[19] Behr O., Brinkmann R., Korner-Nievergelt F., Nagy M., Niermann I., Reich M., Simon R., Rüter S. (2016). Reduktion des Kollisionsrisikos von Fledermäusen an Onshore-Windenergieanlagen (RENEBAT II). Umwelt Raum.

[20] Medina I., Newton E., Kearney M.R., Mulder R.A., Porter W.P., Stuart-Fox D. (2018). Reflection of near-infrared light confers thermal protection in birds. Nat. Commun..

[21] Rublee E., Rabaud V., Konolige K., Bradski G. ORB: An efficient alternative to SIFT or SURF. Proceedings of the 2011 International Conference on Computer Vision.

[22] Davies E.R. (2012). Computer and Machine Vision: Theory, Algorithms, Practicalities.

[23] Stojnić V., Risojević V., Muštra M., Jovanović V., Filipi J., Kezić N., Babić Z. (2021). A method for detection of small moving objects in UAV videos. Remote Sens..

[24] Davies D., Palmer P.L., Mirmehdi M., Carter J.N., Nixon M.S. (1998). Detection and Tracking of Very Small Low Contrast Objects. Proceedings of the British Machine Vision Conference 1998.

[25] Wang Z., Bovik A.C., Sheikh H.R., Simoncelli E.P. (2004). Image quality assessment: From error visibility to structural similarity. IEEE Trans. Image Process..

[26] Sunrise, Sunset and Daylength. https://www.timeanddate.com/sun/germany/stuttgart.

[27] Grodsky S.M., Behr M.J., Gendler A., Drake D., Dieterle B.D., Rudd R.J., Walrath N.L. (2011). Investigating the causes of death for wind turbine-associated bat fatalities. J. Mammal..

[28] Brinkmann R., Behr O., Korner-Nievergelt F. (2011). Entwicklung von Methoden zur Untersuchung und Reduktion des Kollisionsrisikos von Fledermäusen an Onshore-Windenergieanlagen. Umwelt Raum.

[29] Mathews F., Swindells M., Goodhead R., August T.A., Hardman P., Linton D.M., Hosken D.J. (2013). Effectiveness of search dogs compared with human observers in locating bat carcasses at wind-turbine sites: A blinded randomized trial. Wildl. Soc. Bull..

[30] Arnett E.B. (2006). A preliminary evaluation on the use of dogs to recover bat fatalities at wind energy facilities. Wildl. Soc. Bull..

[31] Domínguez del Valle J., Cervantes Peralta F., Jaquero Arjona M.I. (2020). Factors affecting carcass detection at wind farms using dogs and human searchers. J. Appl. Ecol..

